# The future of pharmaceuticals: strategic foresight in an era of uncertainty

**DOI:** 10.1080/20523211.2026.2724624

**Published:** 2026-09-14

**Authors:** Kateryna Ostrovska

**Affiliations:** Health Policy and Systems Strategist, Foresight Practitioner (Freelance consultant), Dnipro, Ukraine

If you don't take the time to think about the future, you are almost guaranteed a future of constraint, instead of a future of choice.

– Sheryl Connelly, former Corporate Chief Futurist

The International Monetary Fund’s quarterly analysis of uncertainty trends across more than 140 countries shows that uncertainty has generally risen over the past decade. The World Uncertainty Index increased sharply from mid-2024, peaked in late 2025, and then moderated, while remaining above earlier levels. Similar patterns are evident in policy and trade uncertainty, as reflected in the World Policy Uncertainty Index and the World Trade Uncertainty Index, respectively (World Uncertainty Index, 2026).

At the same time, the World Economic Forum (2026), in its assessment of global risks, defined as the possibility of developments or events that could cause significant damage to GDP, human populations, or natural resources, emphasises a shift from a relatively stable risk environment toward one characterised by greater volatility, interdependence, and uncertainty. This marks the tipping point at which conventional risk management reaches its limits (Spitz & Desbiey, 2025), making deliberate and systematic engagement with uncertainty essential for organisations seeking to prepare more effectively for the future (Rohrbeck & Kum, 2018).

The pharmaceutical sector is no exception, operating within a macro-environment that is continually shaped by external forces (Siddiqui, 2021). These forces may range from early indicators of change, or weak signals (van Veen & Ortt, 2021), to emerging trends that signal broader directions of change (Fasnacht & Schlosser, 2025), and long-standing megatrends that typically evolve over years or decades and arise at the intersection of multiple trends (Naughtin et al., 2024), including interlinkages among megatrends (Marmier et al., 2022). While differing in the scale of their potential impact, these forces may also be classified according to the nature of the underlying factors – political, economic, social, technological, environmental, and legal (PESTEL) (Siddiqui, 2021).

Simultaneously, the pharmaceutical industry remains an integral component of the broader health system, interacting with key system elements, including governance and management arrangements, financing structures, the health workforce, health information technologies, and service delivery models (Plsek & Greenhalgh, 2001; WHO, 2007). Due to the inherent complexity and adaptability of these systems, such interactions can produce feedback mechanisms and emergent patterns, with causal relationships rarely unfolding in linear, unidirectional, or fully predictable ways. Over time, system trajectories may also be influenced by path dependencies (Kreienkamp & Pegram, 2020; Long et al., 2018; McCoy & Allotey, 2021; Plsek & Greenhalgh, 2001; Snowden & Boone, 2007).

Consequently, rather than attempting to define a single predicted future for pharmaceutical products and technologies, these characteristics underscore the value of complementing forecasting with exploratory research into a wider range of possible futures (Taleb et al., 2022; Tetlock et al., 2014; Tetlock & Gardner, 2016). This perspective aligns with Dator’s ‘Laws’ of the Future, which hold that the future is neither singular nor predetermined (Dator et al., 2015).

Such sector-specific strategic foresight would involve observing and interpreting factors that may shape future change across the pharmaceutical value chain, while identifying the actions organisations should take in response (Iden et al., 2017). Additionally, scenario development can help account for the broader macro-environment, sectoral dynamics, and the specific circumstances of the organisation in question (Ramirez & Wilkinson, 2016).

## Know your animals: a typology of uncertainty

In pursuit of this futures research, spanning a time horizon to 2036, the widely used Rumsfeld Matrix provides a useful starting point for classifying different types of events (Stucky et al., 2026). Based on the extent to which uncertainties are knowable, elements of this typology can be mapped onto corresponding metaphors in the conceptual futurist’s menagerie (Table 1).

**Table 1.** Typology of uncertainty, illustrative drivers of change, and strategic responses in pharmaceutical policy and practice.

Type of uncertainty	Animal metaphor	Characteristics	Examples of drivers of change shaping the futures of therapeutics and pharmaceutical policy	Appropriate strategic response
Known knowns	Gray Rhino (Wucker, 2016)	Highly visible, high-impact potentialities whose likelihood and significance are broadly recognised but which are underestimated, deprioritized, or insufficiently addressed.	Population ageing and rising medicine demand; increasing burden of chronic diseases and multimorbidity; healthcare digitisation and patient empowerment; future pandemic preparedness gaps and antimicrobial resistance; climate change and environmental degradation.	Future-oriented strategic planning, including scenario planning; adaptive policymaking; comprehensive risk assessment and management; contingency planning; and regularly reviewed and updated crisis-management protocols (Wucker, 2016).
Known unknowns	Black Elephant (Hine, 2010)	Highly probable, high-impact potentialities recognised or anticipated by experts but insufficiently acknowledged, accepted, or addressed by decision-makers and other stakeholders.	Emerging infections; dependence on geographically concentrated pharmaceutical manufacturing; geopolitical fragmentation of pharmaceutical supply chains.	Participatory foresight, horizon scanning, scenario planning, contingency planning, policy stress testing, adaptive governance.
Unknown knowns	Black Jellyfish (Sardar & Sweeney, 2016)	Seemingly ordinary phenomena that can escalate through feedback, interconnection, and increasing complexity, producing disproportionate or systemic impacts.	Confluence of regulatory changes and restrictive economic measures	Systems thinking and futures literacy training, participatory foresight, horizon scanning, scenario planning, contingency planning, policy stress testing, adaptive governance.
Unknown unknowns	Black Swan (Taleb, 2007)	High-impact events that are not perceptible or articulated in advance, even by experts, and therefore appear as unexpected outliers when they occur.	Radical technological breakthroughs and unprecedented biological threats affecting pharmaceutical production and distribution; unforeseen geopolitical or societal shocks, natural and man-made disasters with cascading pharmaceutical consequences.	Resilience building; redundancy and diversification; horizon scanning and scenario planning; preparedness for rapid response and recovery.

Source: Author’s synthesis based on the Rumsfeld Matrix (Stucky et al., 2026), the menagerie of postnormal potentialities (Sardar & Sweeney, 2016), the Gray Rhino concept (Wucker, 2016) and the PESTEL framework (Siddiqui, 2021).

*Known knowns,* for instance, correspond to ‘*gray rhinos’* (Wucker, 2016): highly visible, high-impact potentialities whose likelihood and significance are recognised but which may nevertheless be underestimated, deprioritized, or insufficiently addressed. *Known unknowns*, by contrast, correspond to ‘*black elephants’*: highly probable, high-impact potentialities recognised or anticipated by experts but insufficiently acknowledged, accepted, or addressed by decision-makers and other stakeholders (Hine, 2010).

*Unknown knowns*, represented by ‘*black jellyfish’* (Sardar & Sweeney, 2016), refer to seemingly ordinary phenomena whose interactions, feedback, or escalation can generate disproportionate or systemic impacts, making their significance difficult to recognise in advance. Finally, *unknown unknowns*, represented by ‘black swans’ (Taleb, 2007), are high-impact events that are not perceptible or articulated in advance, even by experts, and therefore emerge as unexpected outliers.

To begin with the *gray rhinos* (Table 1), several recognised but insufficiently addressed social drivers have the potential to accelerate pharmaceutical innovation and reconceptualize pharmacy as an institution and the pharmacist’s role. These include demographic shifts and changing profiles of healthcare service users (Babar & Almarsdottir, 2015). The average number of years lived in poor health is projected to increase from 10.2 years in 2025–11.4 years by 2050, while the share of the global population aged over 65 is expected to rise from approximately one in ten today to one in six by 2050 (Beauvais et al., 2026). At the same time, existing models of care remain insufficiently responsive to sex – and gender-specific needs, contributing to the under-screening, underdiagnosis, and undertreatment of women (World Economic Forum & McKinsey Health Institute, 2024).

These pressures also point to substantial opportunities for system transformation. Every US$1 invested in health is estimated to generate US$4 in economic value, while concerted efforts to address preventable disease could reduce the global disease burden by 35%, saving an estimated 33 million lives and preventing 460 million years of poor health (Beauvais et al., 2026). Addressing critical gaps in women’s healthcare alone could prevent an estimated 70,000 heart attacks, heart failure episodes, strokes, and other adverse events annually in the United States. Globally, a stronger emphasis on prevention could generate a three – to six-fold return on investment (World Economic Forum & McKinsey Health Institute, 2026).

On the technological and regulatory side, the growing availability of digital technologies is shifting the balance of influence between normatively constrained healthcare professionals and increasingly empowered healthcare consumers, who are becoming active drivers of experimentation and innovation (Fisher et al., 2016). Past outbreaks, including SARS, MERS, and COVID-19, demonstrate that even widely recognised pandemic risks can leave health systems exposed, exemplifying the *gray rhino* manifestation (Wucker, 2016).

Adding an environmental dimension, the rapidly expanding global biotechnology and pharmaceutical sector is itself a significant contributor to climate change. Although the largest companies are reducing emissions as they pursue net-zero targets, the sector’s overall carbon intensity continues to rise, driven in part by smaller actors across the value chain (My Green Lab, 2025).

Turning to the *black elephants*, the emergence of new diseases provides a fitting illustration: their occurrence can be anticipated, yet their clinical manifestations and specific consequences cannot be defined in advance (Spitz & Desbiey, 2025). Europe’s profound dependence on global pharmaceutical sourcing, particularly from China and India (Cavusgil et al., 2025; European Fine Chemicals Group, 2022), provides another example of a vulnerability that has long been recognised within the industry but insufficiently acknowledged at the European policy level. As a result, Europe’s share of global active pharmaceutical ingredient (API) manufacturing declined from 53% in 2000 to 25% in 2022 (Medicines for Europe, 2022).

The picture becomes even more concerning when raw and starting materials, good manufacturing practice intermediates, and APIs are considered together: nearly three-quarters of these pharmaceutical value-chain elements are manufactured outside the European Union (EU) (Medicines for Europe, 2022). Only recently the EU moved to address this strategic dependency through the Critical Medicines Act, which seeks to diversify supply chains, strengthen strategic reserves of essential medicines, and safeguard patient access to treatment (von der Leyen, 2024).

Whether these measures will be sufficient to strengthen the EU production capacity and reduce strategic dependencies remains uncertain. Efforts to increase or re-shore production face several structural constraints, including underinvestment in manufacturing capacity and processes, weak financial and commercial incentives for large upfront investments, high labour costs, low profit margins, market uncertainty, comparatively high compliance costs and administrative burdens, and procurement practices that have historically prioritised price over security of supply (Advancy, 2024; European Court of Auditors, 2023; EFPIA, 2025).

Applied to the pharmaceutical policy and practice, the *black jellyfish*, a confluence of otherwise recognisable regulatory and economic developments, illustrates how interactions among drivers of change may produce effects that are difficult to anticipate. Deloitte identified persistent digital transformation, evolving regulatory practices, pricing pressures, and shifting geopolitical dynamics as key forces transforming the life sciences sector (Lyons et al., 2025).

In the regulatory domain, the EU Artificial Intelligence Act, Corporate Sustainability Reporting Directive, European Health Data Space, China’s volume-based procurement programme, and ongoing restructuring within the U.S. Food and Drug Administration and Department of Health and Human Services are developing in parallel. At the same time, inflation, tariffs, and supply-chain disruptions are adding further economic pressure (Lyons et al., 2025). Additionally, tariffs, for example, are increasingly perceived not simply as a tax or customs duty but as a sourcing challenge (Hakim et al., 2026). The significance of this confluence lies in how these technological, political, economic and regulatory developments may interact with existing pharmaceutical supply-chain dependencies and medicine shortages, producing effects that are disproportionate to any individual driver.

The Russian full-scale invasion of Ukraine and the Israel – Palestine conflict illustrate how geopolitical shocks can generate far-reaching and difficult-to-anticipate consequences across the pharmaceutical and research ecosystems. Their impacts have included damage to pharmaceutical value-creation infrastructure, disruptions to clinical trials and pharmaceutical supply chains, and wider pressures on the global medicines market (Bondarenko et al., 2023; Soliman, 2025). Although some may argue that the conflicts themselves were not entirely unforeseeable, the scale, duration, and cascading nature of their consequences may nevertheless assume *black swan* characteristics. Their effects may reverberate over the longer term, reshaping investment decisions, supply-chain strategies, and the geographic distribution of pharmaceutical research and production (Bobier et al., 2026; Soliman, 2025).

Artificial intelligence (AI) is widely recognised as a major technological driver of change in the life sciences, with agentic AI attracting growing attention. Yet adoption remains uneven: a recent survey of life science executives found that only one in five reported having fully deployed AI across their organisations (Lyons et al., 2025). In R&D specifically, just 11% of respondents to Deloitte’s 2025 R&D Lab of the Future Survey reported having established a predictive laboratory environment grounded in data-driven decision-making (Morgan et al., 2025), while fewer than 10% reported achieving substantial returns on their AI investments (Lyons et al., 2025).

Against this backdrop, AI’s existing use in designing proteins, protein complexes, and short genomic sequences (Hayes et al., 2025) suggests that recent advances in generative genomics may retrospectively acquire *black swan* characteristics. AI-guided methods have enabled the design of complete bacteriophages capable of infecting and killing *E. coli*, expanding the possibilities for genome-scale engineering of biological systems (King et al., 2026). These developments heighten the need for regulatory and ethical frameworks, including those governing clinical evidence for phage-based therapies and the adaptation of clinical protocols (Fortaleza et al., 2026). They also raise biosecurity concerns and require appropriate safeguards (Hassan et al., 2026; ScreenDNA, 2026), challenges that may be compounded by fragmented regulatory oversight, particularly in the United States (Johnson et al., 2025).

## Anticipating change, shaping response

Paraphrasing William Gibson’s famous observation that ‘the future is already here – it’s just not very evenly distributed’ (Gibson, 1999), one might say that the future of the pharmaceutical field is already taking shape around us. Its early signs can be traced through familiar creatures from the uncertainty menagerie – *‘gray rhinos,’* ‘*black elephants,’* ‘*black jellyfish,’* and ‘*black swans’* – whose potential implications can be brought onto the radar through systematic anticipation, adaptive decision-making, and the development of organisational capacity to respond to emerging change and uncertainty.

Foresight is not merely a theoretical exercise but is increasingly embedded in practical policy and decision-making. Since 2020, the WHO has incorporated foresight into its global health work to strengthen Member States’ capacity for anticipatory decision-making. This initiative promotes the integration of futures thinking and horizon scanning into strategic health planning, enabling countries to identify emerging developments, strengthen preparedness for future change, and harness the potential of new technologies to address evolving health challenges (WHO, 2026).

At the EU level, strategic foresight has been incorporated into the Better Regulation Toolbox to inform impact assessments and evaluations (European Commission, 2023). In parallel, anticipatory governance capacities have been strengthened across EU institutions, including through the work of the European Commission’s Joint Research Centre and the European Parliamentary Research Service (Woensel, 2024).

The European Centre for Disease Prevention and Control has further applied strategic foresight to public health by examining future challenges in infectious disease prevention and control (European Centre for Disease Prevention and Control, 2025). Similarly, the European Monitoring Centre for Drugs and Drug Addiction (2023) has explored futures of illicit drug use and addiction and examined how interventions and policies might evolve to address emerging challenges across Europe.

National foresight ecosystems likewise contribute to public health policy and planning. For example, Finland’s foresight activities provide projections and alternative scenarios for future healthcare service needs at both the wellbeing services county and national levels through 2040 (Finnish Institute for Health and Welfare, 2026). Similarly, the Dutch Public Health Foresight Study has supported long-term public health planning for more than three decades by providing systematic assessments of future health trends and their implications (National Institute for Public Health and the Environment, 2025).

Foresight also has deeper roots in the institutional use of science, technology, and innovation analysis to identify emerging opportunities and inform policy and innovation in biotechnology and the life sciences (Antunes & Canongia, 2006; Canongia et al., 2004; Pélissier, 2024). Japan’s large-scale Science and Technology Foresight programme, conducted approximately every five years since the 1970s, provides a prominent example. Its 11th exercise combined forecasting and backcasting to develop a conceptual scenario linking a future societal vision with relevant science and technology domains and the societal issues requiring attention (National Institute of Science and Technology Policy, 2019).

Pharmaceutical and biotechnology companies and research institutions are also applying foresight to strategic decision-making. Foresight exercises, including collaborative strategic foresight, have been used to inform R&D priorities and support new product development (Desai, 2014; Li et al., 2022), while scenario-based approaches have been applied to explore the future of clinical trials (Lee et al., 2026).

Medtech may offer an early indication of broader shifts, as the sector increasingly uses foresight-driven approaches that could be adapted across life sciences and pharmaceuticals. For example, medtech companies are incorporating critical uncertainties into scenarios that guide operational decisions and support early-warning and guardrail systems aimed at strengthening organisational resilience. Recent applications include integrating both imposed and anticipated tariffs into weekly financial analyses to evaluate potential effects on future revenues and supply chains (Hakim et al., 2026).

## Summary

Taken together, these developments demonstrate that the future of the pharmaceutical sector is neither predetermined nor amenable to prediction through a single forecast. It is shaped by interacting demographic, technological, regulatory, economic, environmental, and geopolitical forces unfolding across different time horizons and levels of uncertainty. The menagerie of postnormal potentialities provides a useful lens for distinguishing between foreseeable challenges, overlooked or underestimated developments, emergent systemic effects, and genuinely unpredictable disruptions. Across these domains, strategic foresight offers a means of moving beyond reactive risk management toward systematic anticipation, enabling policymakers and organisations to identify emerging changes, explore alternative futures, test assumptions, and strengthen their capacity to make informed and adaptive choices.
